# Supplementary material for: Microscale pH variations during drying of soils and desert biocrusts affect HONO and NH3 emissions
Source: Nat Commun. 2019 Sep 2;10:3944. doi: 10.1038/s41467-019-11956-6 (PMC6718665; doi:10.1038/s41467-019-11956-6)
Supplement: Supplementary file 1 — Supplementary information [file 41467_2019_11956_MOESM1_ESM.pdf]

Supplementary Information for

Microscale pH variation during drying of soils and desert biocrusts affect HONO and NH_3 emissions

by Minsu Kim and Dani Or

Supplementary Figures

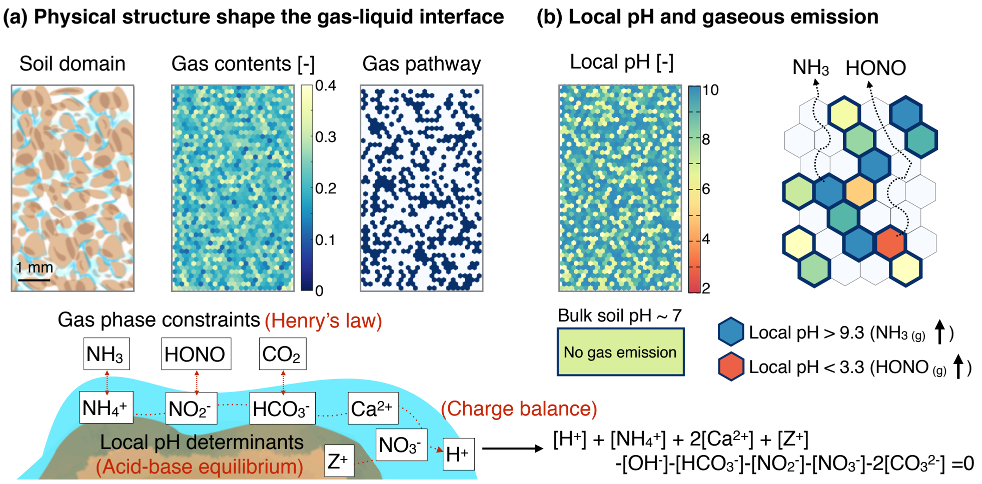

Supplementary Figure 1. An example of the physical domain and the scheme for local pH calculation (a) Physical structure of a soil domain is abstracted and discretised to spatial elements, hexagons, to fill the domain which we termed as a patch. From the invasion percolation of gas, pathways of gaseous compounds are obtained at a certain hydration condition (In this figure, the matric potential was assigned -3 kPa, after the gas phase percolates through the domain). The connectedness of a patch to the atmosphere is given as gas pathway in white. Navy coloured patches indicate patches with air pockets that are disconnected from the atmosphere. The connectedness of gas pathways shapes the interface between soil water and air, thus constrains concentrations of gaseous compounds that affect local pH within the domain. In the model, inorganic carbon and nitrogen are considered and three principles are applied at patch scale (around 10 μm), Henry's equilibria, acid-base equilibria, and local charge balance. (b) By solving diffusion equations and mass transfer between gas-liquid phases, a distribution of local pH at steady state can be calculated. Under the absence of biotic processes, the spatial variability of air-soil water interface drives the spatial heterogeneity of local pH, which cannot be captured from the bulk property, such as soil pH. The soil pH (of the entire domain) is about 7 in the figure which is calculated from the total amount of protons in the domain. When the bulk soil pH is considered, simultaneous emissions of ammonia (NH_3) and nitrous acid (HONO) cannot be predicted at a static hydration condition.

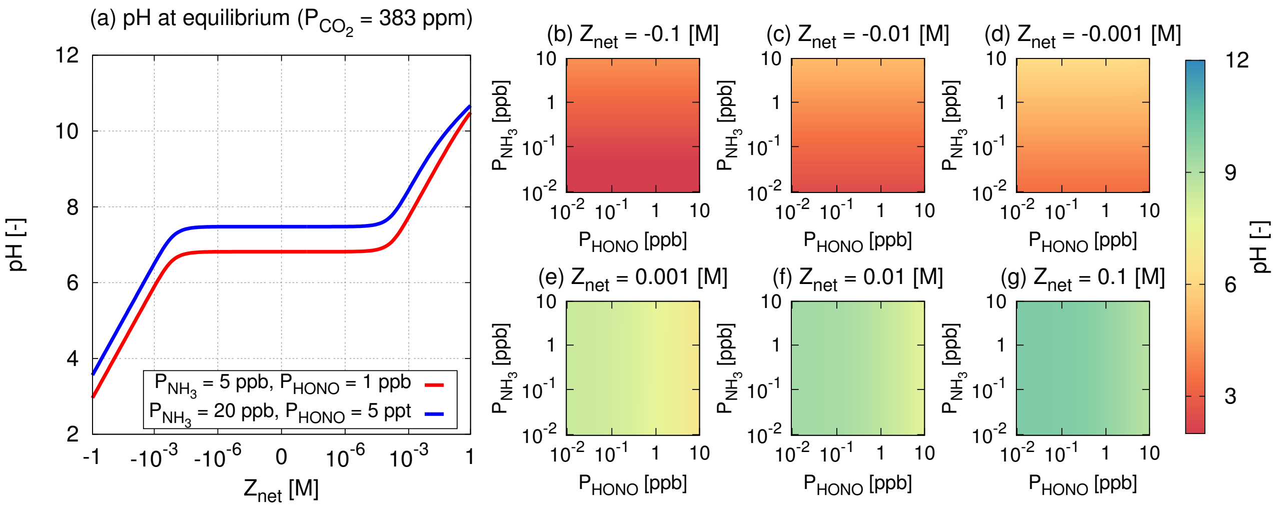

Supplementary Figure 2. Analytic solution of pH (a) Analytic solution of pH at Henry's and acid-base dissociation equilibria as a function of $Z_{net} \equiv 2[Ca^{2+}] + [Z^+] - [NO_3^-]$, which is a net charge of chemical compounds that are not constrained by the air. Under the condition of the constant partial pressure of carbon dioxide (CO_2), 383 ppm, two cases are given as examples, $P_{NH_3} = 5$ ppb, $P_{HONO} = 1$ ppb (red) and $P_{NH_3} = 20$ ppb, $P_{HONO} = 5$ ppt (blue). (b-g) Analytic solution of pH as a function of P_{NH_3} and P_{HONO} for various values of Z_{net}. The results depict that Z_{net} strongly determine the local pH of water film together with partial pressure of pH dependent gaseous compounds. When cations are dominant ($Z_{net} > 0$), the solution indicates alkaline and when anions are dominant ($Z_{net} < 0$), the solution indicates acidic. This indicates that the balance between the chemical environments and the activity of microorganisms (such as production of nitrate, NO_3^-) is crucial for determining local pH of waterfilm on surfaces.

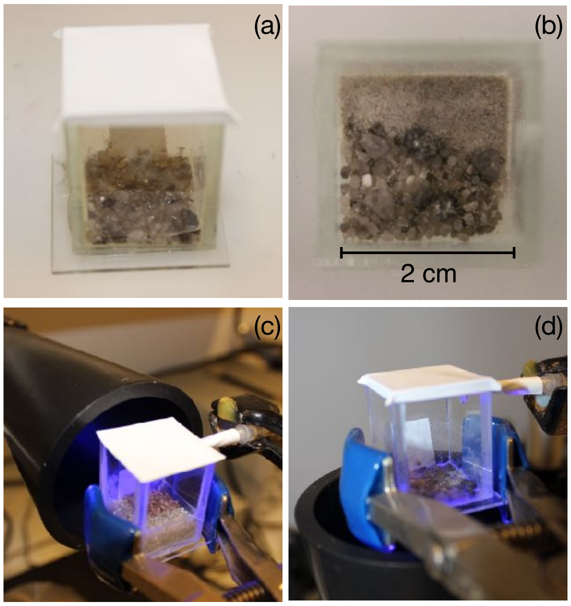

Supplementary Figure 3. Samples used in lab experiments (a) A typical sample glass cuvette (2cm x 2cm x 2cm) filled with quartz sand grains. Sensor foils are mounted on the bottom or one of sides of the cube for horizontal and vertical measurements, respectively. (b) Top view of the sample. Gamma-ray sterilised quartz sand was deliberately distributed in two regions for horizontal measurements. Two distinctive grain size distributions were used for fine texture (0.08 - 0.2 mm) and for coarse texture (0.7 - 3 mm). (c) An example image of vertical measurements. (d) An example image of horizontal measurements.

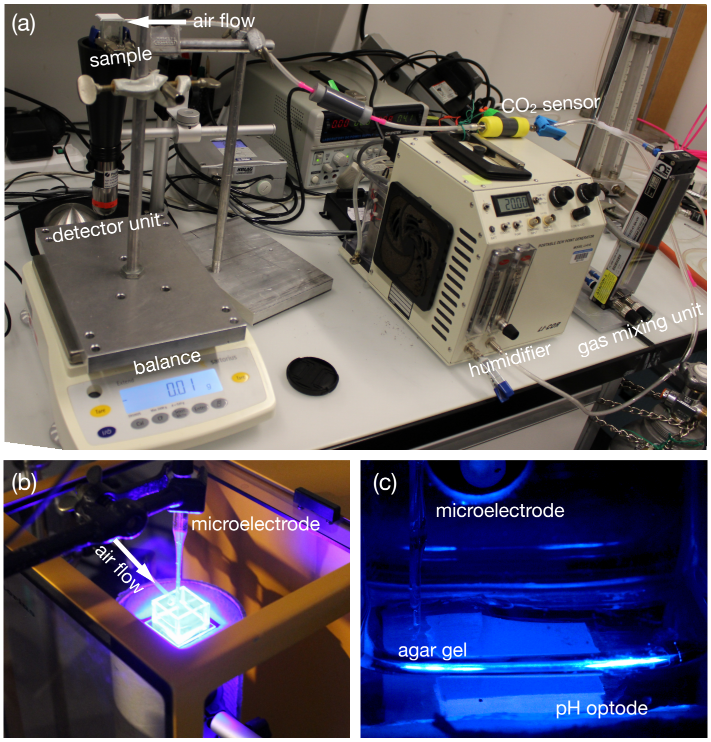

Supplementary Figure 4. Experimental setup (a) From the right side of the image, the amount of carbon dioxide (CO_2) gas in the gas mixture was controlled by the gas mixing unit and the flow rate of the air, together with its humidity and temperature, were controlled with the humidifier. The amount of CO_2 was monitored before it is injected into the glass cuvette sample. During drying the sample using the airflow, the weight of the sample was monitored together with the changes in pH. (b) An image of the simultaneous measurements of pH changes by using a pH optode (on the bottom of the sample) and the microelectrode. The blue LED light is on when the detection unit (the optode camera) captures the image of pH distribution. (c) A close-up image of the glass cuvette sample filled with an agar block in PBS buffer solution.

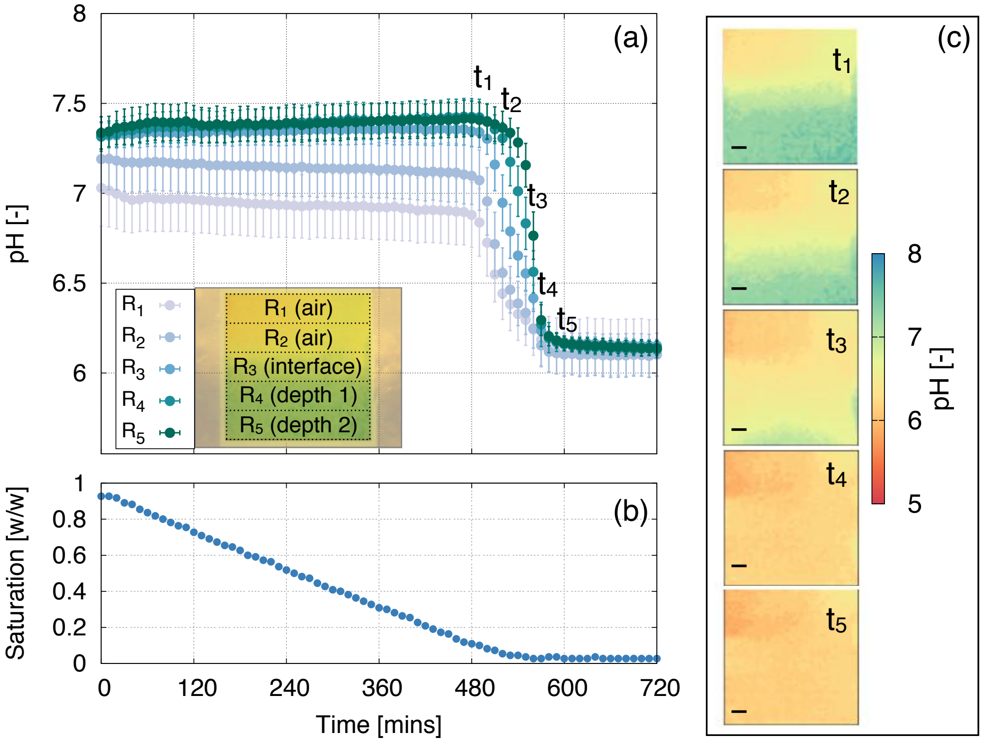

Supplementary Figure 5. Vertical patterns of pH changes The vertical variation indicates that air entry drives acidity of the liquid phase. (a) The response of the optode sensor is analysed for 5 regions, denoted as R_1 to R_5. From top to bottom, regions indicate air, air-soil interface, and soil. Spatially averaged pH values for each region are given during a course of the desiccation. Error bars indicate one standard deviation of spatial distributions. The inset figure shows an overlay of the sample and pH values. (b) Saturation degree of the soil sample is calculated as the weight ratio of water loss while drying to the amount of applied water. (c) Dynamics of pH distributions at the transition are given every 25 mins ($t_1 = 480, \cdots t_5 = 600$ mins). Corresponding time points are marked in figure (a). The scale bar indicates 1mm.

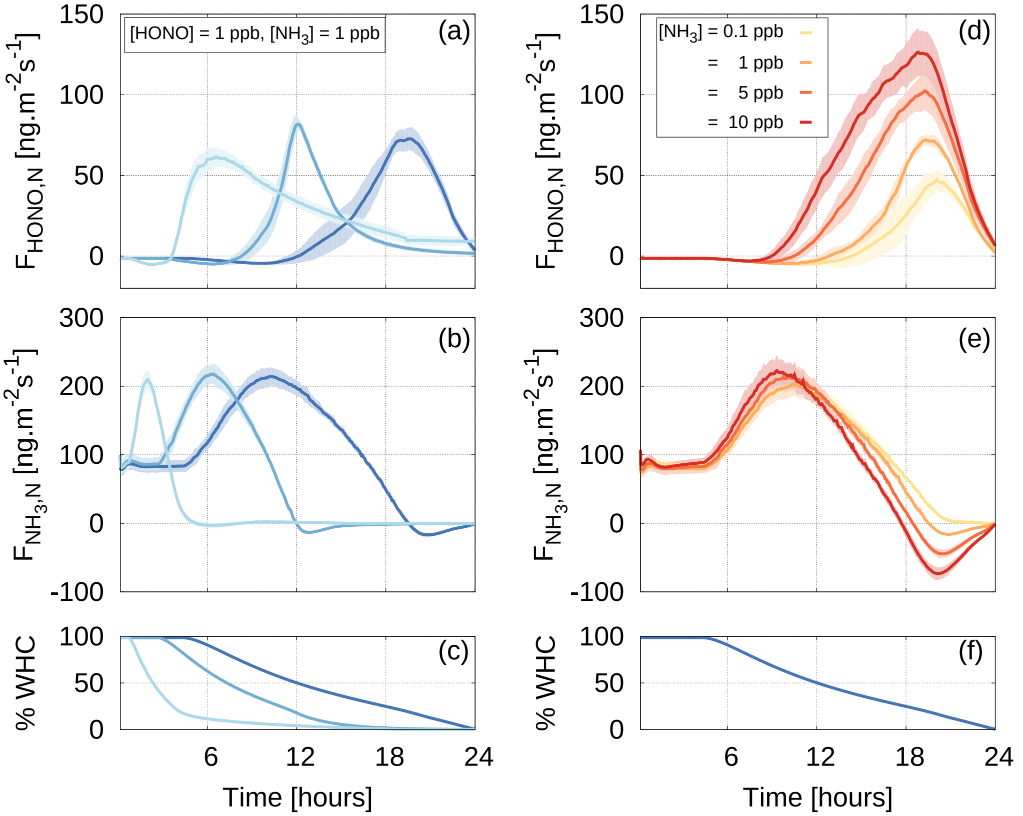

Supplementary Figure 6. Nitrous acid and ammonia emission dynamics Nitrous acid (HONO) and ammonia (NH_3) emissions from simulated desiccation of biocrusts under various conditions. Solid lines are the averages and shaded areas are 1 standard deviation of 8 independent simulations under the same boundary conditions. (a) Dynamics of HONO emissions and (b) NH_3 emissions under (c) three different patterns of simulated drying events. Each drying event is given with the corresponding blue colour code. The stronger blue indicates the slower drying rate, thus the biocrust stays hydrated longer. During these simulations, the mixing ratios of HONO and NH_3 are fixed as 1 ppb and 1 ppb, respectively. Effects of NH_3 input on (d) Dynamics of HONO emissions and (e) NH_3 emissions during (f) a drying event are given with a spectrum of reds, the stronger red indicates higher dry deposition of NH_3. Here, the mixing ratio of HONO was fixed as 1ppb.

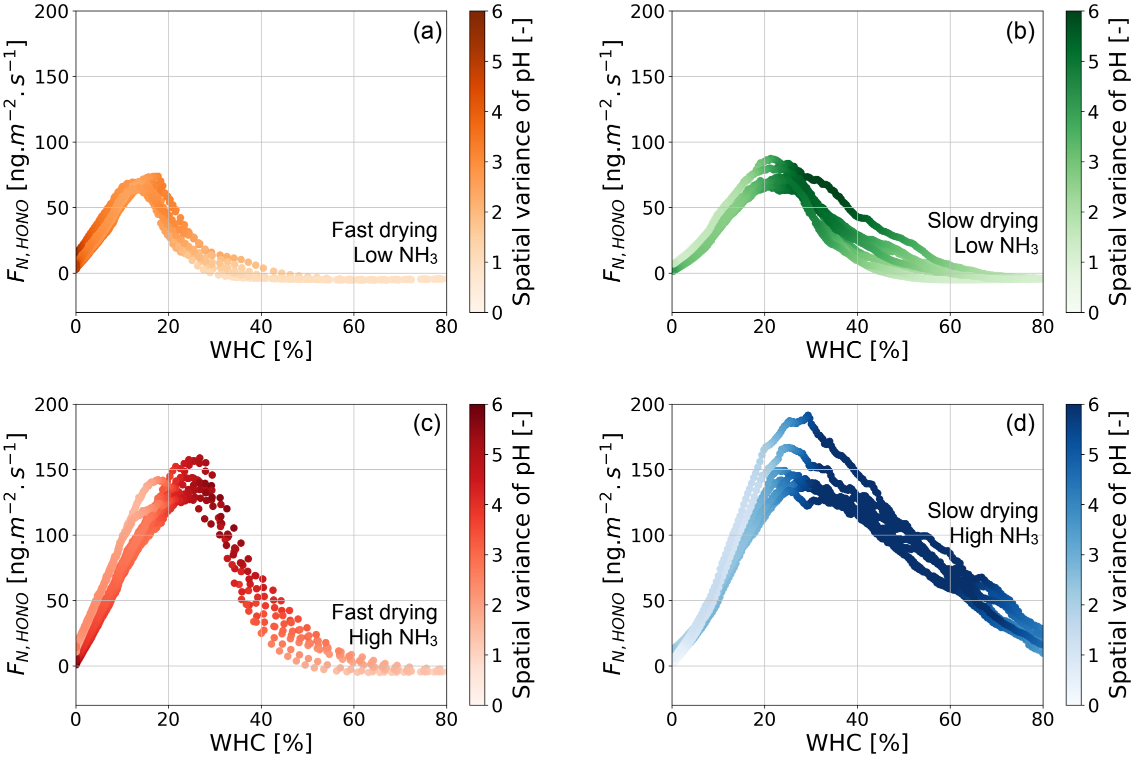

Supplementary Figure 7. Dynamics of nitrous acid emission and spatial variation of pH while drying soils Dynamics of nitrous acid (HONO) emission as a function of hydration conditions (represented as percentage water holding capacity). Here, eight independent simulations under the same boundary conditions were plotted together. Four different conditions correspond to Figure 6 in the main text. The spatial variance of pH is given with a colour map for each condition. (a) Fast drying with low ammonia (NH_3) input, (b) slow drying with low NH_3 input, (c) Fast drying with high NH_3 input, and (d) slow drying with high NH_3 input.
